# Causal association of sex hormone-related traits with Alzheimer’s disease: a multivariable and network Mendelian randomization analysis

**DOI:** 10.3389/fneur.2025.1391182

**Published:** 2025-02-05

**Authors:** Yan Zhang, Zhen-dong Sun, Yu-shen Yang, Wei-dong Fu

**Affiliations:** ^1^Department of Anesthesiology, Zhuzhou Hospital Affiliated to Xiangya School of Medicine, Central South University, Zhuzhou, Hunan, China; ^2^Department of Anesthesiology, The Second Affiliated Hospital of Fujian Medical University, Quanzhou, Fujian Province, China

**Keywords:** Alzheimer’s disease, sex hormone-binding globulin (SHBG), testosterone, neurodegenerative disease, Mendelian randomization

## Abstract

**Background:**

Although studies have demonstrated a correlation between sex hormone-related traits [such as sex hormone binding globulin (SHBG) and testosterone] and Alzheimer’s Disease (AD), the link remains uncertain due to the intricacies of AD pathology. The study aimed to investigate the possible causal link between sex hormone-related traits and AD.

**Methods:**

The authors collected data from extensive genome-wide association studies (GWASs) of European ancestry on sex hormone-related traits and AD. Univariate and multivariate Mendelian randomization (MR) analyses were conducted to explore the possible causal relationship between these traits and AD. We used inverse variance weighted (IVW) MR as the main analysis.

**Results:**

The use of univariate MR-IVW revealed a possible causal relationship between SHBG [ORs (95% CI), 1.005 (1.001–1.009), *p* = 0.006], testosterone [ORs (95% CI), 0.994 (0.989–0.999), *p* = 0.013] and AD in female. There is no evidence of a causal association of SHBG [ORs (95% CI), 1.002 (0.999–1.005)), *p* = 0.237] and testosterone [ORs (95% CI), 1.000 (0.997–1.004), *p* = 0.810] with AD in males. Multivariate MR analysis revealed a possible direct causal connection between SHBG and testosterone in relation to females AD (SHBG-OR (95%CI), 1.005 (1.001–1.009, *p* = 0.023); testosterone-OR (95%CI), 0.995 (0.989–1.000, *p* = 0.049). Bidirectional MR analysis indicated that SHBG has a possible causal effect on testosterone (SHBG on testosterone-OR (95%CI), 1.064 (1.032–1.096), *p* = 0.0001). The results of the network MR analysis suggested that testosterone may act as a mediator in the causal pathway from SHBG to AD.

**Conclusion:**

Our study using the MR methodology indicates that increase of SHBG level and decrease of testosterone level in females are positively linked to an increased risk of developing AD. Importantly, testosterone plays a mediating role in the causal pathway from SHBG to females AD.

## Introduction

Alzheimer’s disease (AD), the most prevalent form of dementia, is an age-related neurodegenerative disease (1). According to Alzheimer’s Disease International, approximately 50 million individuals worldwide were estimated to be living with dementia in 2018, and this figure is projected to exceed 150 million by 2050 (2). AD is one of the leading causes of mortality and morbidity, representing a significant social and economic challenge globally. Numerous risk factors for developing AD have been identified, including gender (2), age, ethnicity, family history and genetics (3), physical activity, smoking and drinking status, body mass index (BMI), diabetes mellitus, and depression. Epidemiological studies indicates that female are more likely to develop AD than male, potentially due to hormonal changes associated with aging.

Sex hormone-related traits, such as sex hormone binding globulin (SHBG) and testosterone, play a crucial role in human brain development, exhibiting neuroprotective effects by maintaining neural function and promoting neuronal survival (4). Recently, the involvement of sex hormone-related traits in the development of AD has gained attention. For example, previous study using small sample sizes have focused on free testosterone levels in male AD patients and age-matched control groups. This research indicates that reduced levels of free testosterone may pose a distinct risk for AD (5). The relationship between SHBG and AD, however, remains ambiguous. A large prospective study indicated that high serum level of SHBG, the primary plasma binding protein of sex hormones (6), was associated with an increased risk of all-cause dementia, including AD, among middle-aged to older women (7). Conversely, a recent MR study by Yeung et al. (8) found no significant association between SHBG and AD. Therefore, a comprehensive analysis of the relationship between sex hormones, SHBG, and AD is imperative.

Mendelian randomization (MR) is a statistical approach that utilizes genotypic instrumental variables (IVs) based on three main hypotheses: a strong association of IVs with exposure factors, no association of IVs with confounders, and that IVs influence outcomes exclusively through exposure (9). These hypotheses facilitate the inference of relationships between exposure factors and outcomes. MR uses genetic variants, such as single nucleotide polymorphisms (SNPs), as IVs. These genetic variants are randomly assigned during gametogenesis, akin to randomization in controlled trials, and remain unaffected by acquired environmental factors (10) (e.g., lifestyle, socioeconomic status). This characteristic allows MR to mitigate the confounding factors that can bias exposure-outcome relationships to a certain extent. In addition, based on the Mendelian law of inheritance, MR only affects the outcome variables through exposure factors, and does not affect the outcome through other ways, which makes the causal inference power stronger than observational research (11). Consequently, MR methodology is gaining increasing traction.

In this study, we utilized the genome-wide association studies (GWAS) database to explore the possible causal relationship between sex hormone-related traits and AD. This approach can facilitate to elucidate the genetic characteristics of AD and provide an innovative perspective for this study of AD, specifically by targeting the regulation of sex hormone-related traits for the prevention and treatment of AD.

## Methods

### Data source

The analysis performed in this research drew on publicly available GWAS summary statistics for a maximum of 425,097 white European individuals from the UK Biobank. Independent SNPs relating to SHBG and testosterone were identified. The sample sizes for SHBG and testosterone in males were 185,221 and 199,569, respectively. For females, the sample sizes were 214,989 and 199,569. The sample size for AD and healthy controls was 26,757 and 283,086 cases, respectively. AD documented in the UK Biobank was diagnosed and determined in electronic health records (EHR)-linked illness records, which includes ICD-10 codes assigned by clinical doctors according to hospital admissions and death registries (12, 13).

### Instrumental variables

We chose SNPs associated with exposure at the genome-wide significance threshold (*p* < 5e-8). We also excluded SNPs with linkage disequilibrium (r^2^ < 0.001 within 10,000 kb) to ensure independence of each IV. We excluded SNPs that may have been associated with the outcome from the IVs (*p* < 0.05) and conducted several sensitivity analyses to test for horizontal pleiotropy. Finally, F-statistics were calculated for each IVs, and only IVs with F-statistics >10 were retained to avoid bias caused by weaker IVs (14). After combining the genetic predictors of SHBG and testosterone, we utilized the “clustering” function to exclude overlapping SNPs (r^2^ > 0.05). The remaining SNPs were utilized for MR analysis.

### Mendelian randomization analysis

All models underwent initial analysis using inverse variance weighting (IVW). The results, including odds ratios (ORs) and 95% confidence intervals (CIs), were presented, which is considered the primary method for assessing causality in MR studies (15). Significant estimates uncovered by IVW were later augmented with two additional MR methods (weighted median and MR-Egger). The weighted median method is described as the median of the weighted empirical density function of the ratio estimates that assigns more weight to the more accurate IVs. This estimate remains robust even when up to half of the data comes from unreliable or less valid instruments (16). The MR-Egger technique is not limited to a zero-slope assumption. Consequently, its causal estimate reflects the dose–response association between genotype and outcome, while accounting for pleiotropic effects (17).

Furthermore, a leave-one-out analysis was designed to test the sensitivity of SNPs. We removed each SNP to perform the results of IVW and then assessed the impact of each SNP on the results. The sensitivity of this SNP was reflected via the fluctuation of the outcomes before and after removing each SNP. Funnel plots were drawn to observe the causal influence of each SNP and to examine whether the outcomes were influenced by potential biases.

Multivariable MR was used to estimate the direct effect of females SHBG and testosterone on AD after adjusting each other, respectively. The multivariable MR IVW was selected as primary analysis. In sensitivity analysis, MR Egger was used to test whether the genetic predictors were acting other than via SHBG and testosterone (directional pleiotropy) indicated by a non-zero intercept.

Additionally, the bidirectional MR analysis was used to investigate the possible causal link between SHBG and testosterone, which includes two univariable MR tests. Finally, if causal association between SHBG and testosterone was observed via bidirectional MR and the results from the univariate MR are statistically significant, we further investigated the causal mediating effect through network MR analysis (18, 19).

## Results

### Characteristics of genetic variants

**Figure 1:**
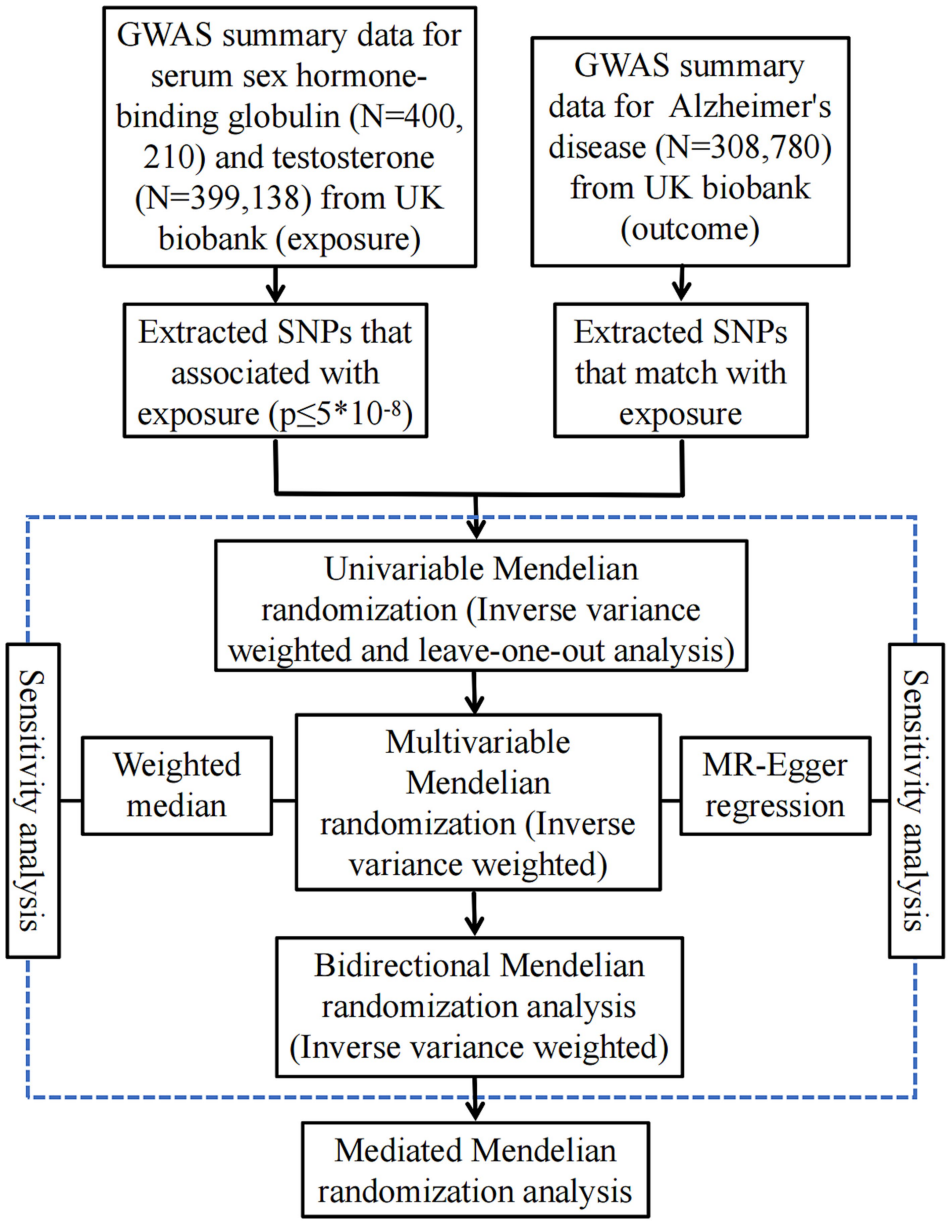
A flow diagram of the process in this MR analysis. SNPs, single nucleotide polymorphism; GWAS, genome-wide association studies.

Using the genome-wide significance cutoff of 5 × 10^−8^ for screening, we identified a total of 195 SNPs associated with females SHBG, 104 SNPs that were linked to female testosterone, 201 SNPs relative to male SHBG and 167 SNPs pertaining to male testosterone. It is worth noting that the F statistic values of the IVs were all >10, suggesting that there was a robust relationship of IVs with AD and that there was no weak bias. Genetic associations with sex hormone-related traits can be found in Table 1. A flowchart of the study design is shown in Figure 1.

**Table 1:** Summary information of the data sets.

Traits	Data source	Year	Sample size	Case	Control	Population
Female SHBG	UK Biobank	2020	214,989	NA	NA	European
Female testosterone	UK Biobank	2020	199,569	NA	NA	European
Male SHBG	UK Biobank	2020	185,221	NA	NA	European
Male testosterone	UK Biobank	2020	199,569	NA	NA	European
Alzheimer's disease	UK Biobank	2017	308,780	26,757	283,086	European

### Univariable Mendelian randomization

Using IVs to investigate the possible causal link between AD and sex hormone-related traits, the results indicate a possible causal association of SHBG [ORs (95% CI), 1.005 (1.001–1.009), *p* = 0.006; Table 2] and testosterone [ORs (95% CI), 0.994 (0.989–0.999), *p* = 0.013; Table 2] with AD in females. However, there is no evidence of a causal association of SHBG [ORs (95% CI), 1.002 (0.999–1.005), *p* = 0.237; Table 3] and testosterone [ORs (95% CI), 1.000 (0.997–1.004), *p* = 0.810; Table 3] with AD in males.

**Table 2:** Associations of genetically predicted female SHBG and testosterone in Alzheimer's disease using univariable MR in the UK Biobank.

Exposure	Outcome	MR method	Number of SNPs	Beta	SE	OR (95% CI)	*P*-value
		Inverse variance weighted	175	0.005	0.002	1.005 (1.001–1.009)	**0.006**
		Weighted median	175	0.005	0.003	1.004 (0.998–1.011)	0.153
SHBG	AD	MR Egger	175	0.003	0.004	1.003 (0.996–1.010)	0.41
		Simple mode	175	0.008	0.007	1.008 (0.994–1.023)	0.248
		Weighted mode	175	0.005	0.004	1.005 (0.997–1.012)	0.224
		Inverse variance weighted	104	0.006	0.002	0.994 (0.989–0.999)	**0.013**
		Weighted median	104	0.001	0.004	0.999 (0.991–1.007)	0.843
Testosterone	AD	MR Egger	104	0.001	0.005	0.999 (0.990–1.008)	0.827
		Simple mode	104	0.015	0.01	0.985 (0.967–1.004)	0.13
		Weighted mode	104	0.001	0.005	0.999 (0.990–1.008)	0.867

AD, Alzheimer’s disease; MR, Mendelian randomization; SHBG, sex hormone-binding globulin; SNPs, single nucleotide polymorphisms. Bold values indicate statistical significance, p < 0.05.

**Table 3:** Associations of genetically predicted male SHBG and testosterone in Alzheimer's disease using univariable MR in the UK Biobank.

Exposure	Outcome	MR method	Number of SNPs	Beta	SE	OR (95% CI)	*P*-value
SHBG	AD	Inverse variance weighted	174	0.002	0.002	1.002 (0.999–1.005)	0.237
		Weighted median	174	0.001	0.003	1.001 (0.996–1.006)	0.739
		MR Egger	174	0.003	0.003	0.997 (0.991–1.003)	0.338
		Simple mode	174	0.003	0.006	1.003 (0.992–1.015)	0.595
		Weighted mode	174	0.001	0.003	0.999 (0.994–1.005)	0.85
Testosterone	AD	Inverse variance weighted	140	0	0.002	1.000 (0.997–1.004)	0.81
		Weighted median	140	0.003	0.003	1.003 (0.997–1.009)	0.361
		MR Egger	140	0.001	0.003	0.999 (0.993–1.006)	0.847
		Simple mode	140	0.001	0.007	1.001 (0.988–1.014)	0.875
		Weighted mode	140	0.002	0.003	1.002 (0.995–1.009)	0.546

AD, Alzheimer’s disease; MR, Mendelian randomization; SHBG, sex hormone-binding globulin; SNPs, single nucleotide polymorphisms.

### Multivariable Mendelian randomization

We made an attempt to control for pleiotropic pathways that might arise from the association between different sex hormone-related traits and established a multivariable MR model including SHBG, testosterone jointly as exposures for AD. The significant link of females SHBG and testosterone to AD observed in the univariable MR remained significant [SHBG-OR (95% CI): 1.005 (1.001–1.009), *p* = 0.023; testosterone-OR (95% CI): 0.995 (0.989–1.000), *p* = 0.049] after adjusting for each other. Details were presented in Table 4.

**Table 4:** Associations of genetically predicted female SHBG and testosterone in Alzheimer's disease using multivariable MR (IVW).

Exposure	Outcome	MR method	nSNPs	Beta	SE	OR (95% CI)	*P*-value
Testosterone	Alzheimer's disease	MR-IVW	73	0.005	0.003	0.995 (0.989–1.000)	0.049
SHBG	Alzheimer's disease	MR-IVW	143	0.005	0.002	1.005 (1.001–1.009)	0.023

MR, Mendelian randomization; SHBG, sex hormone-binding globulin; SNPs, single nucleotide polymorphisms.

### Bidirectional MR analysis and mediated MR analysis

Bidirectional MR analysis with IVW indicated a causal possible effect of SHBG on testosterone [SHBG on testosterone: 1.064 (1.032–1.096), *p* = 0.0001], with a positive correlation. However, testosterone had an insignificant effect on SHBG [testosterone on SHBG: OR (95% CI), 1.048 (0.918–1.196), *p* = 0.490] (Table 5). The results of the network MR analysis indicated that testosterone may act as a mediator in the causal pathway of SHBG on AD, and accounts for 6.2% of the overall impact of SHBG on AD (the effect of SHBG on testosterone is 0.062, the effect of testosterone on AD is 0.005, so the mediating effect of testosterone is equal to 0.062 × 0.005 = 0.00031, the mediated proportion was the mediating effect of testosterone/the total effect of SHBG on AD = 0.00031/0.005 = 6.2%) (see Table 6).

**Table 5:** The Bidirectional MR analysis results between female SHBG and testosterone.

Exposure	Outcome	MR method	nSNP	Beta	SE	OR (95% CI)	*P*-value	MR-Egger intercept *p*-value
SHBG	Testosterone	IVW	191	0.062	0.015	1.064 (1.032–1.096)	**0**	0.562
SHBG	Testosterone	Weighted median	191	0.054	0.016	1.056 (1.024–1.089)	**0.001**	
SHBG	Testosterone	MR-Egger	191	0.075	0.028	1.078 (1.021–1.139)	**0.008**	
Testosterone	SHBG	IVW	112	0.047	0.067	1.048 (0.918–1.196)	0.49	0.864
Testosterone	SHBG	Weighted median	112	−0.035	0.02	0.966 (0.929–1.005)	0.085	
Testosterone	SHBG	MR-Egger	112	0.028	0.13	1.028 (0.797–1.325)	0.832	

MR, Mendelian randomization; SHBG, sex hormone-binding globulin; SNP, single nucleotide polymorphisms. Bold values indicate statistical significance, *p* < 0.05.

**Table 6:** The mediation effect of SHBG on Alzheimer's disease via testosterone.

Beta (XZ)	Beta (ZY)	Beta (XY)	b	Mediated proportion
0.062	0.005	0.005	0.00031	0.062

### Sensitivity analysis

#### Univariable Mendelian randomization

Sensitivity analysis was conducted using MR-Egger regression and Weighted median. Their results corresponded in direction with the estimates of the IVW. The results from MR-Egger analysis revealed *p* = 0.410 for SHBG, *p* = 0.827 for testosterone in females, *p* = 0.338 for SHBG, *p* = 0.847 for testosterone in males. All results with *p* > 0.05 indicated no horizontal pleiotropy, suggesting the results of the present study are stable and reliable.

On the other hand, the results from leave-one-out analysis were also consistent with the results using all SNPs. No SNPs showed strong effect on the results (Supplementary Figures 1–4). The funnel plot showed the basically symmetrical causality of a single SNP, suggesting that the results are highly unlikely to be influenced by potential bias (Supplementary Figures 5–8). Totally, the results from sensitivity analysis demonstrated the stability and reliability of our results.

#### Multivariable Mendelian randomization

A large *p-*value for the intercept term (*p-*value 0.181) was also observed in the multivariable MR-Egger analysis, suggesting low possibility of horizontal pleiotropy (Supplementary Table 1). In addition, the point estimate of the slope corresponded in direction with the estimates of the multivariable MR IVW.

#### Bidirectional MR analysis

In the bidirectional MR analysis, these estimates from MR-Egger regression and Weighted median were also similar in direction with the results of the IVW (Table 3). It is worth noting that the MR-Egger analysis yielded a large *p-*value for the intercept term (*p* = 0.56 for of SHBG and *p* = 0.86 for testosterone), identifying the probability of horizontal pleiotropy is low.

## Discussion

Our study applied MR to explore the bidirectional possible causal connection between traits related to sex hormones and AD. The univariate MR outcomes indicated that AD in females exhibited a correlation with SHBG and testosterone, with SHBG positively correlated with AD, while testosterone demonstrated a negative correlation. Additionally, there was no casual correlation for SHBG and testosterone with AD in males. Sensitivity analyses further confirmed this correlation. We subsequently conducted multivariate MR analyses on testosterone and SHBG in females AD patients, revealing a possible causal association between testosterone and SHBG on AD. The results were in agreement with univariate MR. Our bidirectional and network MR analyses demonstrated that testosterone serves as a significant mediator in the causal pathway from SHBG to AD. In summary, higher SHBG levels are associated with increased risk of developing AD, potentially due to decreased testosterone levels.

In both univariate and multivariate MR analyses, SHBG was positively linked to AD in female, whereas testosterone exhibited a negative association with AD in female, corroborating findings from certain previous studies. For instance, Muller et al. (20) discovered that elevated levels of SHBG correlated with reduced cognitive performance and an augmented risk of developing AD, and an overall increased susceptibility to dementia. Additionally, for every standard deviation increase in serum SHBG concentrations, the risk of developing dementia escalated by between 20 and 30% across a mean follow-up time of 5.2 years (20). Moreover, various epidemiological studies have highlighted higher SHBG levels in females AD patients in contrast to healthy controls (21–24). There is limited research examining the association between testosterone and AD in female. Our study identified both a negative correlation and a possible causal link between the two variables. This could be due to SHBG blocking testosterone from binding to its receptor, leading to a reduction in bioavailable testosterone. Thus, any positive correlation between SHBG and AD in male is likely to be mediated by available testosterone rather than a direct effect.

Numerous studies have reported on the association between SHBG, testosterone, and AD. A study using the UK Biobank has revealed that there is a link between the development of dementia and AD in 159,411 men aged 50–73 years (25) with lower testosterone and higher serum SHBG levels. This study is the largest of its kind investigating the relationship between dementia and testosterone. However, we only observed strong associations between SHBG, testosterone, and AD in female, and the effects of SHBG and testosterone on AD were not significant in male. These discrepancies may arise from the limited sample sizes in standard observational studies and the possible impact of various confounding factors.

Follicle-stimulating hormone (FSH) and luteinizing hormone (LH) are gonadotropins (GnH) secreted by gonadotropic cells in the anterior pituitary gland. These hormones regulate reproductive function through a complex feedback mechanism. Webber et al. (26) proposed that increased GnH concentration, rather than decreased estrogen production, may contribute to the elevated risk of AD following menopause. Preclinical experiments have revealed that LH impairs cognition through direct action on hippocampal LH receptors (LHCGRs) (27, 28), while FSH has been observed to accelerate amyloid *β* and Tau deposition and impair cognitive function in mice with AD (29). In a small sample of male, LH was also associated with plasma amyloid-β (30, 31). Nerattini et al. (32) demonstrated that elevated serum gonadotropin levels, particularly FSH, were associated with an increased risk of biomarkers of AD in middle-aged female. This suggests a potential correlation between GnH and cognitive decline.

Gender and age are significant risk factors for AD, with evidence suggesting it is more prevalent in female. Female tend to live an average of 4.5 years longer than male and constitute the majority of individuals aged over 85 years globally (33). Age is the most critical risk factor for AD, however, assessing actual risks for individuals of the same age can be challenging. After age, genetic factors are the second most important risk factor associated with AD. This finding is not surprising, as parents with dementia have a heightened risk of transmitting AD to their offspring (34, 35). Evidence suggests that disease expression can differ across generations, with children of affected parents experiencing later onset of symptoms (AAO) (34–36). We selected a study sample whose mother was diagnosed with AD to investigate this phenomenon. Thus, genetic factors may have had some influence on our results as well.

Despite the reduction in the possibility of confounding factors, the study has limitations. First, the data utilized were sourced from the GWAS database, which primarily reflects European populations. Consequently, the applicability of these results to other ethnic populations may be limited, as gene–environment interaction patterns can vary across different ethnic groups. Second, the potential overlap of participants in the GWAS dataset for the exposures and outcomes under investigation remains unclear. Third, except gender, this study did not perform a detailed analysis to eliminate the influence from other confounders on our conclusions. Finally, it should be noted that sex hormones and gonadotrophins interact with each other, mainly affecting the hypothalamic–pituitary-gonadal axis. However, this study mainly focused on testosterone and SHBG, and the other hormones were not studied in depth. Further comprehensive studies on the association between other sex hormones, including gonadotrophins, and AD may be needed in the future. Although we explored the potential mediating role of testosterone in AD, the conversion of testosterone in the brain mainly involves two pathways, including the conversion to dihydrotestosterone by 5α-reductase and the conversion to estradiol by aromatase. We are still not sure whether testosterone or its transforming substances exert their effects. Therefore, the findings of this study necessitate validation through additional clinical and basic research.

Taken together, our study using the MR methodology indicates that increase of SHBG level and decrease of testosterone level in females are positively linked to an increased risk of developing AD. Importantly, testosterone plays a mediating role in the causal pathway from SHBG to females AD. However, these causal associations were not observed in male AD, which might tend to support the conclusion that the conversion of testosterone to estradiol is likely the pathway through which increased SHBG and lower testosterone increases the risk of female AD.

## Data Availability

The original contributions presented in the study are included in the article/Supplementary material, further inquiries can be directed to the corresponding author.
