# Supplementary figures and images for: Causal association of sex hormone-related traits with Alzheimer’s disease: a multivariable and network Mendelian randomization analysis
Source: Front Neurol. 2025 Feb 5;16:1391182. doi: 10.3389/fneur.2025.1391182 (PMC11835684; doi:10.3389/fneur.2025.1391182)

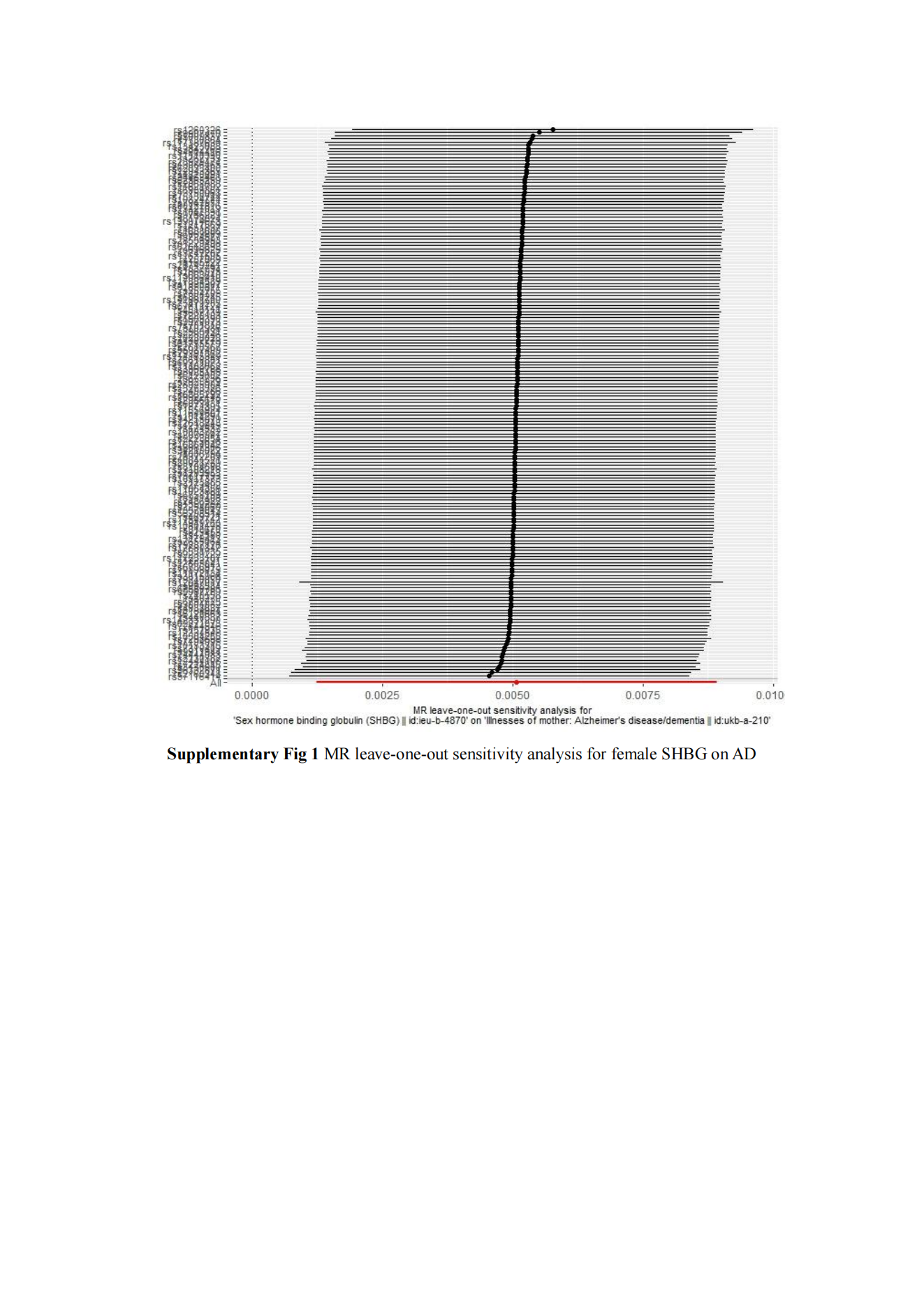

Supplement: Supplementary file 2 [file Image_1.tif]

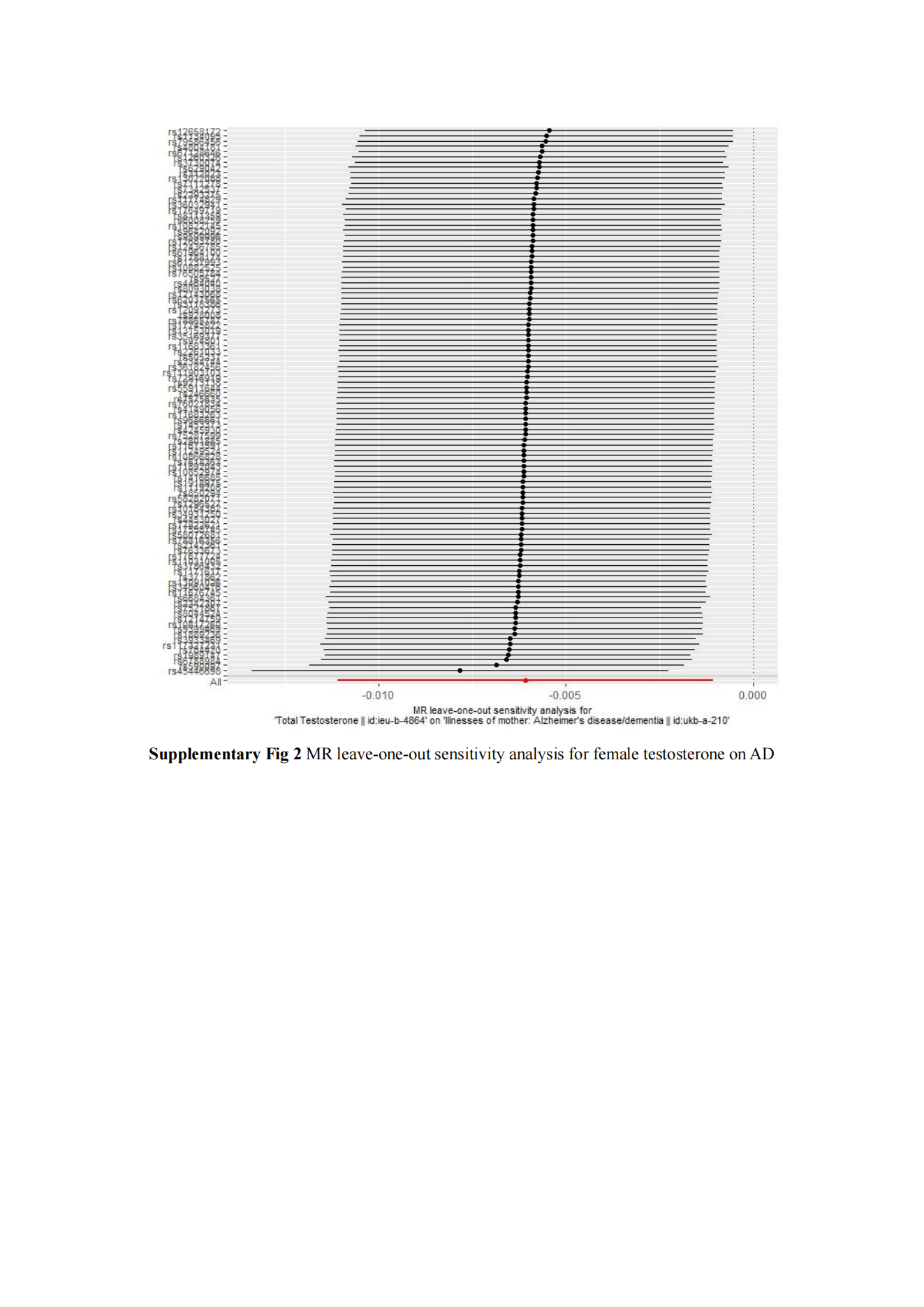

Supplement: Supplementary file 3 [file Image_2.tif]

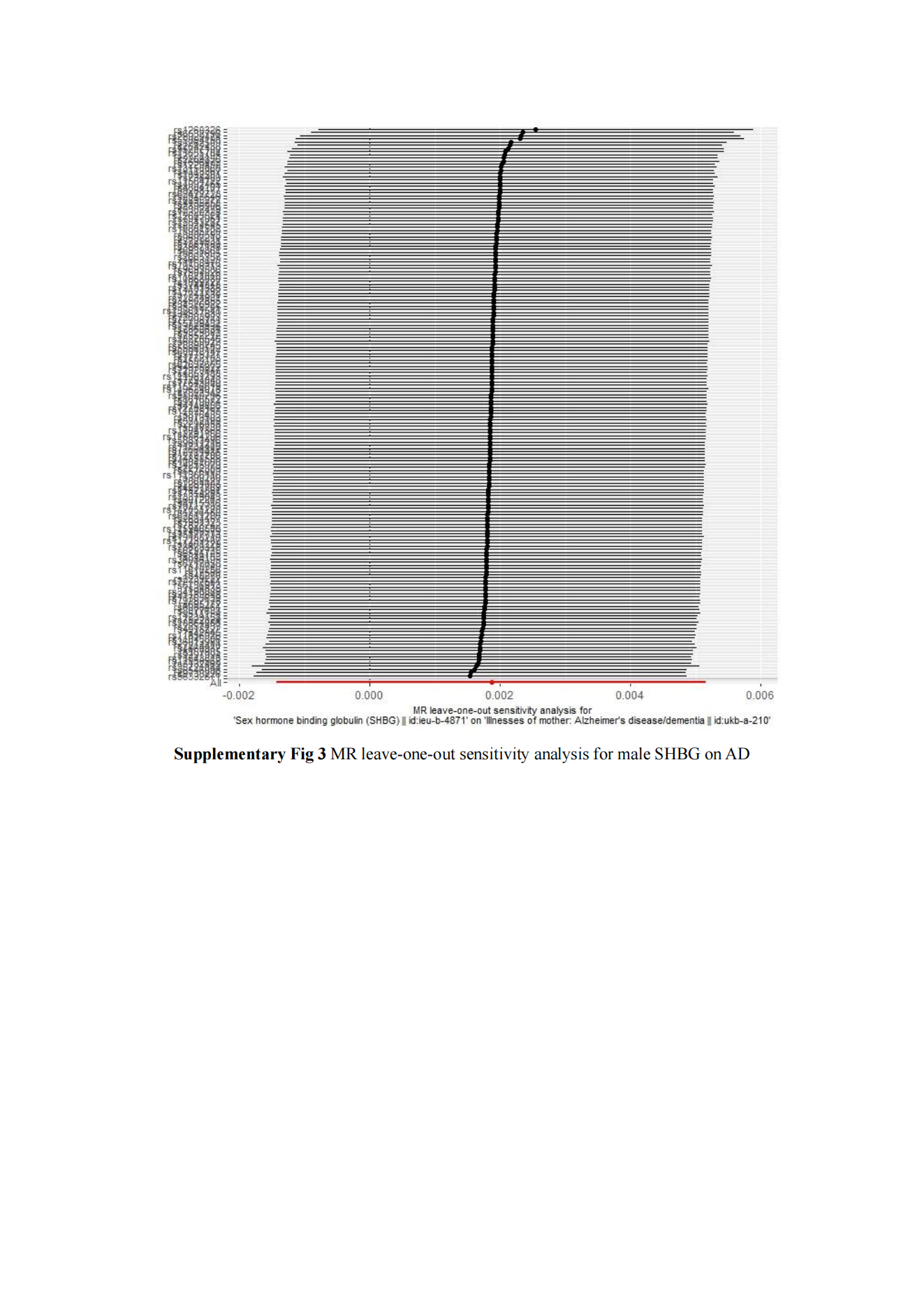

Supplement: Supplementary file 4 [file Image_3.tif]
